# Different Forms of ER Stress in Chondrocytes Result in Short Stature Disorders and Degenerative Cartilage Diseases: New Insights by Cartilage-Specific ERp57 Knockout Mice

**DOI:** 10.1155/2018/8421394

**Published:** 2018-12-17

**Authors:** Yvonne Rellmann, Rita Dreier

**Affiliations:** Institute of Physiological Chemistry and Pathobiochemistry, Waldeyerstraße 15, 48149 Münster, Germany

## Abstract

Cartilage is essential for skeletal development by endochondral ossification. The only cell type within the tissue, the chondrocyte, is responsible for the production of macromolecules for the extracellular matrix (ECM). Before proteins and proteoglycans are secreted, they undergo posttranslational modification and folding in the endoplasmic reticulum (ER). However, the ER folding capacity in the chondrocytes has to be balanced with physiological parameters like energy and oxygen levels. Specific cellular conditions, e.g., a high protein demand, or pathologic situations disrupt ER homeostasis and lead to the accumulation of poorly folded or misfolded proteins. This state is called ER stress and induces a cellular quality control system, the unfolded protein response (UPR), to restore homeostasis. Different mouse models with ER stress in chondrocytes display comparable skeletal phenotypes representing chondrodysplasias. Therefore, ER stress itself seems to be involved in the pathogenesis of these diseases. It is remarkable that chondrodysplasias with a comparable phenotype arise independent from the sources of ER stress, which are as follows: (1) mutations in ECM proteins leading to aggregation, (2) deficiencies in ER chaperones, (3) mutations in UPR signaling factors, or (4) deficiencies in the degradation of aggregated proteins. In any case, the resulting UPR substantially impairs ECM protein synthesis, chondrocyte proliferation, and/or differentiation or regulation of autophagy and apoptosis. Notably, chondrodysplasias arise no matter if single or multiple events are affected. We analyzed cartilage-specific ERp57 knockout mice and demonstrated that the deficiency of this single protein disulfide isomerase, which is responsible for formation of disulfide bridges in ECM glycoproteins, is sufficient to induce ER stress and to cause an ER stress-related bone phenotype. These mice therefore qualify as a novel model for the analysis of ER stress in chondrocytes. They give new insights in ER stress-related short stature disorders and enable the analysis of ER stress in other cartilage diseases, such as osteoarthritis.

## 1. Cartilage Enables Skeletal Development, Bone Growth, and Diarthrodial Joint Function

Cartilage is a connective tissue with essential functions in embryonic development and throughout life. During bone development by endochondral ossification, cartilaginous templates of future bones are formed and later gradually replaced by bone [1, 2]. The bone formation starts with the generation of cartilage condensations, consisting of prechondrogenic mesenchymal cells. These cells differentiate into chondrocytes, produce a cartilage-specific ECM, and build bone templates (= bone anlagen), in which the central chondrocytes start to proliferate and progressively differentiate into metabolically highly active hypertrophic chondrocytes. During proliferation and hypertrophic differentiation, chondrocytes produce large amounts of extracellular proteins that form structural components of the ECM or act as local growth factors. Through a variety of these growth factors, the chondrocytes trigger the differentiation of osteoblasts from the surrounding periosteum to form the bone collar [3]. In addition, vascular invasion is initiated and brings osteoblasts and osteoclasts into this so-called primary ossification center. These cells replace the cartilage by bone through removal of cartilaginous extracellular matrix and deposition of newly formed trabecular bone. Most hypertrophic chondrocytes die by apoptosis. However, recently, it was proven that some of the osteoblasts arise from hypertrophic chondrocytes by transdifferentiation [4]. All processes recur in the secondary ossification centers in both epiphyses of the long bones. Following ossification in the primary and secondary ossification centers, cartilaginous tissue remains on the bone surfaces as articular cartilage, where it is responsible for frictionless movement of the joints. Cartilage also remains in the growth plates between the ossification centers, where it is responsible for long bone growth until the growth plate fuses during puberty. Lengthening of a bone is dependent on proliferation and maturation of chondrocytes and the increasing production and secretion of ECM molecules by the chondrocytes. However, the largest contribution comes from a dramatic increase in the volume of hypertrophic chondrocytes in the growth plate as they undergo terminal differentiation [5]. Any imbalance between proliferation and hypertrophy can lead to skeletal defects and in particular to chondrodysplasias [6]. Chondrodysplasias comprise several hundred distinct forms of skeletal diseases from severe disorders that are perinatal lethal to milder conditions that are recognized postnatally [7]. The latter are characterized by a disproportionate short stature, eye abnormalities, cleft palate, and hearing loss [8]. In addition to skeletal development and bone growth, cartilage is involved in the maintenance and function of diarthrodial joints.

## 2. A Proper ER Function Is a Prerequisite for Effective Protein Synthesis and Secretion by Chondrocytes

To build and maintain cartilage, a proper function of the endoplasmic reticulum (ER) is essential in chondrocytes, as they are responsible for the production of large amounts of ECM proteins during skeletal development and growth. Due to avascularity of cartilage, the secretory chondrocytes experience a variety of stresses, such as low oxygen tension and limited nutrient conditions [9, 10], and consequently, the protein folding capacity in the cells has to be balanced with physiological parameters like energy and oxygen levels [11]. However, different cellular conditions, e.g., phases of high protein demand or pathologic situations, prevent ER homeostasis and lead to the accumulation of poorly folded proteins. This physiological or pathological state is called ER stress and induces a cellular quality control system, the so-called unfolded protein response (UPR), an adaptive mechanism to cope with ER stress to restore homeostasis [12].

**Figure 1:**
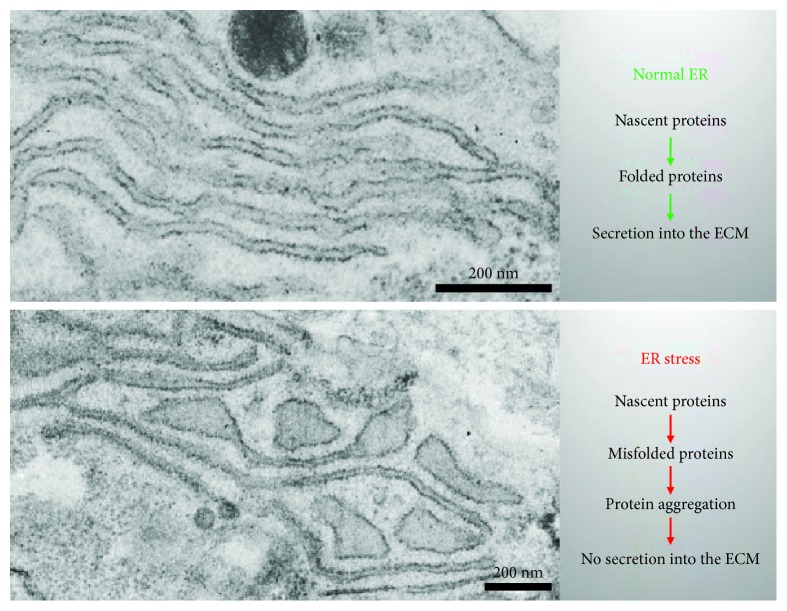
ER stress induces morphological and functional changes in chondrocytes. Normal chondrocytes produce large amounts of cartilage components. Before secretion into the ECM, these proteins undergo posttranslational modification and folding in the ER. If these processes fail, ER stress arises and misfolded proteins aggregate in the ER. This leads to a dilation of ER cisternae and a diminished protein secretion into the ECM.

Prior to their secretion, all proteins destined not only for the extracellular space but also for the plasma membrane or for secretory compartments undergo posttranslational modification, folding, and maturation in the rough ER (Figure 1) [13]. For this purpose, the lumen of the ER contains resident molecular chaperones, protein disulfide isomerases (PDIs), and folding factors that multiply the rate of protein folding. The folding complexes are active in a specific environment of high Ca^2+^ concentration and oxidizing conditions [12, 14]. The chaperones and folding enzymes can be assigned to different protein families: (1) members of the heat shock family (e.g., BiP, GRP94), (2) serpins (e.g., HSP47), (3) lectins (e.g., calreticulin, calnexin, and EDEM), (4) oxidoreductases or protein disulfide isomerases (e.g., PDI, ERp57), and (5) peptidyl-prolyl cis/trans isomerases (cyclophilins, FK506-binding proteins, and parvulin-like peptidyl-prolyl cis/trans isomerases) [14–16]. Initially, the proteins to be folded are targeted to the ER by hydrophobic signal sequences that are cotranslationally recognized by signal recognition particles. After transition through a translocon complex in the ER membrane, the signal peptide is cleaved off by signal peptidases in the ER lumen and the nascent polypeptide is posttranslationally modified, e.g., by the oligosaccharyltransferase which is responsible for N-linked glycosylation. Attachment of carbohydrate moieties, called glycans, to asparagine within the Asn-X-Ser/Thr consensus sequence enhances the intrinsic solubility of nascent polypeptides during folding, potentially by masking hydrophobic patches, but also allows critical interactions with the lectin chaperones calnexin and calreticulin [17].

Via particular domains at their N-termini, the lectins calnexin and calreticulin specifically bind monoglycosylated N-linked glycans on the nascent proteins, after these were attached by the oligosaccharyltransferase and trimmed by glycosidases I and II and ER mannosidases [18]. The N-termini of calnexin and calreticulin in addition bind the PDI ERp57, assisting in folding by disulfide exchange reactions. Like other protein disulfide isomerases in the ER, ERp57 is responsible for correct disulfide bridge formation. After a first round of folding, calnexin or calreticulin releases the protein and glucosidase II removes the final glucose molecule from its glycan, thereby inhibiting the binding of lectin chaperones again. However, until the folding process is not completed, a uridine diphosphate-glucose-glycoprotein transferase adds a new glucose molecule to the glycan again, and the protein enters the calnexin/calreticulin cycle for a second time allowing another round of folding. Such glycosylation-folding reglycosylation cycles continue until the native conformation is finally achieved or proteins aggregate due to misfolding [12, 19]. This review focusses on ERp57, that is, as a part of the calnexin/calreticulin cycle, mainly engaged in folding of glycoproteins with unstructured disulfide-rich domains [20, 21]. An overview about these and other distinct functional roles of ERp57 in various cellular compartments playing a role under physiological and pathological conditions is given elsewhere [22].

Correctly folded proteins move via vesicular transport to the Golgi apparatus. There are additional modifications such as O-glycosylation occur, and sorting of the proteins into different kinds of vesicles is established to enable a further transport to different cellular compartments or secretion into the extracellular space. In case of incorrect folding of ECM proteins or protein overload in the ER, unfolded or misfolded proteins accumulate in the ER and subsequently activate the UPR. This complex quality control system leads to a general stop of cellular protein synthesis, an increased production of additional chaperones and other folding proteins, and to the degradation of aggregated proteins by ER-associated degradation (ERAD type I) or autophagy (ERAD type II). ERAD type II represents an autophagic pathway in which soluble and insoluble misfolded proteins are incorporated in autophagosomes, which then fuse with lysosomes. ERAD type I targets soluble misfolded proteins only. These are polyubiquitinylated and translocated into the cytosol, where they are degraded in the proteasome [23]. However, when the combined efforts of UPR and ERAD do not readjust cellular homeostasis, cell death by apoptosis is initiated, in order to enable general tissue homeostasis. [24].

## 3. The UPR Initiates a Stepwise Rescue System for ER-Stressed Cells

The adaptive UPR comprises three parallel signaling pathways starting from ER stress sensor proteins located in the ER membrane: ATF6*α* (activating transcription factor 6 alpha), IRE1*α* (inositol-requiring enzyme 1 alpha), and PERK (protein kinase RNA-like endoplasmic reticulum kinase) [25–27]. At the luminal side of the ER membrane, BiP (immunoglobulin heavy-chain-binding protein), also known as glucose-regulated protein 78 (GRP78), binds to these sensor proteins and keeps them inactive. Upon binding of BiP to unfolded or misfolded proteins accumulating in the ER, BiP is released from the ER stress sensors, which trigger the UPR signaling pathways. On the first route, BiP-free ATF6*α* traffics to the Golgi apparatus, where it is processed by the site 1 and site 2 proteases (S1P and S2P). The released ATF6*α* fragment acts as a transcription factor, enters the nucleus, and induces UPR genes encoding additional chaperones or initiators of ERAD [28]. On the second pathway, BiP-free IRE1*α* is activated by oligomerization and autophosphorylation [29]. Active IRE1*α* degrades certain mRNAs through regulated IRE1-dependent decay (RIDD) [30] and induces splicing of the transcription factor XBP1 (X-box-binding protein 1). The spliced transcription factor XBP1s (XBP1_spliced_) then directly activates gene expression for folding proteins and quality control mechanisms in the ER. On the third route, oligomerized and autophosphorylated PERK acts as a kinase on eIF2a (eukaryotic translation initiation factor 2A) and thereby stops global transcription, thus reducing the overall protein synthesis and decreasing the load of unfolded proteins in the ER [31]. However, due to preferential translation of mRNAs containing short open reading frames in the 5′ UTRs, the amount of transcription factor ATF4 is increased. ATF4 positively regulates the expression of UPR genes that are involved in amino acid metabolism, antioxidant response, folding, and regulation of autophagy and apoptosis. Examples of such ATF4-induced genes are CHOP (C/EBP homologous protein) and GADD34 (growth arrest and DNA damage-inducible 34) [32].

## 4. ER Stress in Cartilage Is Important under Physiological and Pathological Conditions

One should consider that ER stress or UPR signaling pathways play a crucial role in chondrocytes in phases of high protein synthesis, e.g., during bone development by endochondral ossification. As cartilage is a nonvascularized tissue, low energy levels and hypoxic conditions prevail. ER stress, therefore, is essential for normal differentiation and hypertrophic maturation of chondrocytes under these tough, but physiological conditions [33, 34].

In addition, ER stress is triggered by pathological conditions, such as metabolic dysfunction, Ca^2+^ ion imbalances, and expression of mutant proteins, or inducible by specific drugs. Under all circumstances, the direct consequence of ER stress is the initiation of UPR signaling in order to return to cellular homeostasis. However, this is not always possible, and thus, cellular imbalances occur that lead to ER stress-related pathological outcomes. Due to unresolved ER stress in chondrocytes, diseases of the skeletal system, such as chondrodysplasias, arise.

## 5. Various Mouse Models with ER Stress in Chondrocytes Display Phenotypes Resembling Skeletal Diseases Associated with Growth Plate Abnormalities and Dwarfism

Several whole-body knockout mouse models demonstrate the general necessity of a proper protein folding in the ER for developmental processes, organ function, and cellular homeostasis. Homozygous deletion of ER chaperones such as calreticulin, BiP, GRP94, ERp57, or UDP-glucose-glycoprotein glucosyltransferase results in embryonic lethality [14, 35]. Thus, the function of these proteins is exclusive and essential for embryonic development. Here, we focus on the impact of chondrocytes to the development and growth of long bones. Different actions of these cells are important for endochondral ossification, such as the finely tuned proliferation and maturation, the raising production and secretion of specific ECM molecules, the increase in the volume of hypertrophic chondrocytes, and the exact regulation of chondrocyte death by apoptosis at the lower end of the epiphyseal plate. If one or more of these processes fail, skeletal development is impaired and short stature diseases, like chondrodysplasias, may develop.

In order to specifically analyze the role of ER stress in chondrocytes and its relevance to chondrodysplasias, more and more mouse models with cartilage-specific changes in protein folding have been developed (Table 1) [36, 37]. From these mice, one can deduce that prolonged ER stress, e.g., due to a mutation of an ECM protein initiating poor folding and aggregation in the ER, is a pathogenic mechanism behind short stature diseases like metaphyseal chondrodysplasia type Schmid (MCDS), multiple epiphyseal dysplasia (MED), or pseudoachondrodysplasia (PSACH). Similarly, mice with mutations in proteins of the ER folding machinery [38], mutations in UPR signaling factors [39, 40], or mutations in proteins of the secretory and degradative pathways eliminating aggregated proteins display related skeletal phenotypes. This substantiates that ER stress acts as a pathogenic factor in chondrodysplasias.

To get a well-defined overview about mouse models with ER stress in chondrocytes, one should discriminate between (1) transgenic mice with mutations in genes encoding ECM proteins, (2) transgenic mice with mutations in genes encoding exogenous proteins that are normally not expressed in cartilage, (3) mice with a knockout of genes encoding proteins of the ER folding machinery, (4) mice with a knockout of genes of UPR signaling factors, (5) mice with a knockout of proteins involved in the degradation of aggregated proteins, and (6) mice with a knockout of proteins essential for protein trafficking and secretion.

### 5.1. Transgenic Mice with Mutations in Genes Encoding Cartilage ECM Proteins

The extracellular matrix of cartilage is composed of a set of self-assembled secreted macromolecules that form a dynamic network of fibrillar and nonfibrillar structures. The key macromolecules of cartilage ECM are collagens II, IX, and XI, forming collagen fibrils and thus are mainly responsible for the tensile strength; proteoglycans, primarily aggrecan, responsible for the osmotic swelling and elastic properties; noncollageneous glycoproteins such as COMP and matrilins, connecting various ECM components; and hyaluronan, providing compression strength, lubrication, and hydration within the cartilaginous ECM [41]. Due to mutations in genes encoding such ECM proteins, misfolding may occur during its synthesis, which initiates ER stress. The following mouse models demonstrate that ER stress seems to be critically involved in the development of skeletal diseases, as all mice display chondrodysplasia-like phenotypes, no matter which deficiency they have.

#### 5.1.1. Mouse Model of Chondrodysplasia Associated with a Mutation in the Col2a1 Gene (p.Gly1170Ser in Col2a1)

Mutations in the *α*1 chain of procollagen type II initiate chondrodysplasias of different severity from lethal to mild forms [42]. Such disease-inducing mutations often occur in the triple-helical domain (Gly-X-Y domain) of collagen II alpha 1 chains and initiate intracellular retention of the targeted protein with induction of ER stress and activation of UPR signaling [37]. One prominent example is a knockin mouse model harboring a *col2a1*p.Gly1170Ser mutation, in which the growth plate develops abnormally because chondrocytes undergo apoptosis before hypertrophy. This leads to the disappearance of hypertrophic zones. The detailed investigation of this mouse model suggested that this early chondrocyte death is related to the ER stress-UPR-apoptosis cascade and that this is the main cause of the p.Gly1170Ser-induced chondrodysplasia in mice and men [43].

#### 5.1.2. Mouse Model of Spondyloepiphyseal Dysplasia (SED) Associated with a Arg992Cys (p.Arg1192Cys) Mutation in the Col2a1 Gene

Another class of mutations within the human Col2a1 gene resulting in skeletal abnormalities is a single base substitution that converts codons for arginine in the Y position of the Gly-X-Y domain to codons for cysteine [44–46]. The most interesting is the SED model associated with an Arg992Cys (p.Arg1192Cys) substitution [45, 46]. These mice reveal ER stress and an altered linear bone growth. The growth plates display a disturbed columnar organization of chondrocytes, an altered collagenous matrix, an atypical cell polarization with unusual organization of primary cilia, and a reduced chondrocyte proliferation. Remarkably, this phenotype can be rescued by switching off the expression of mutated collagen II only during embryonic development or in newborn mice but not later, suggesting that possible therapies in human diseases with this mutation must be applied at prenatal or early postnatal stages in order to be successful [46]. Recently, the impact of arginine-to-cysteine mutations in collagen II on protein secretion and cell survival was described elsewhere [47].

#### 5.1.3. Mouse Model of MED (p.Val194Asp in MATN-3)

MED comprises a range of genetically and phenotypically heterogeneous skeletal dysplasias characterized by disorganized endochondral ossification of the epiphyses of long bones and early-onset osteoarthritis in large weight-bearing joints. Autosomal dominant forms of MED can result from mutations in genes encoding type IX collagen, oligomeric cartilage protein (COMP), and matrilin-3 [36]. The MED-causing mutant proteins are structurally unrelated, but all three comprise perifibrillar constituents and most likely form the structural basis for the mutual interaction of the cartilage fibrils with the extrafibrillar matrix [48]. In addition, the mutations lead at least to partial retention of the affected protein in the ER. Leighton et al. generated a murine model of MED by introducing a specific human disease-causing mutation (p.Val194Asp) into mouse matrilin-3 [49]. Homozygous mice of this genotype develop a progressive chondrodysplasia with weight loss and short-limbed dwarfism. Mutant matrilin-3 is retained within the rough ER of chondrocytes, and the aggregated proteins with aberrant disulfide bonding [50] initiate an unfolded protein response, with upregulation of the UPR marker proteins BiP and calreticulin. The mice display disorganized growth plates with reduced proliferation. In addition, spatially increased apoptosis of chondrocytes occurs. However, whether this chondrocyte death is directly linked to ER stress is unresolved yet, as CHOP expression is not correspondingly augmented [36, 51].

#### 5.1.4. Mouse Models of PSACH (p.Thr583Met and p.D469del in COMP)

Mutations in the cartilage oligomeric protein (COMP) can also cause PSACH. PSACH is a more severe skeletal dysplasia, characterized by a marked short stature, a deformation of the legs, and ligamentous laxity [52]. For a detailed analysis of PSACH, different mouse models were used. One harbors a single point mutation (Thr583Met) in the C-terminal domain of COMP [53] which in patients results in a mild form of the disease with typical radiographic features and waddling gait, but normal or only mild short stature [54]. Mutant mice are normal at birth, but grow slower than their wild-type littermates and also develop a mild short-limbed dwarfism. The chondrocyte columns in the growth plates of these mice are poorly organized. Mutant COMP, however, is secreted into the extracellular matrix, but it is dislocated along with several COMP-binding proteins. Although mutant COMP is not retained within the rough ER, an unfolded protein response with upregulated expression of BiP, calreticulin, phosphorylated eIF2*α*, and processed ATF6 is initiated. Chondrocyte proliferation is significantly reduced, while apoptosis is both generally increased and spatially dysregulated. By 16 months of age, mutant animals exhibit severe degeneration of articular cartilage, which is consistent with early-onset osteoarthritis seen in PSACH patients [55]. A second transgenic mouse model carries the most frequent mutation in humans, the deletion p.D469del [56]. In these mice, both wild-type and mutant COMP were detected throughout the growth plate. Mutant molecules were restricted to the pericellular matrix, while wild-type COMP showed a uniform distribution throughout the extracellular matrix. Mice expressing the mutant transgene showed a slight gender-specific growth retardation. In mutant animals, the columnar organization in the growth plate was disturbed, proteoglycans were lost, and improperly formed collagen fibrils were observed. In some chondrocytes, the ER was dilated, most probably due to an impaired secretion of mutant COMP similar to that observed in patients. Later in development, the growth plate was irregularly shaped and prematurely invaded by bony tissue [56]. A third mouse model harbors the D469del COMP mutation as a knockin and was generated by homologous recombination [57]. Most phenotypic characteristics were similar to the transgenic mouse, but in contrast, the knockin mouse showed no canonical UPR signaling although proteins aggregated in the ER. Instead, gene profiles of oxidative stress, cell cycle, apoptosis, and NF-*κ*B signaling changed, suggesting the involvement of UPR-independent stress pathways [57].

#### 5.1.5. Mouse Model of MCDS (13del in COL10a1)

MCDS is a dominant disease caused by mutations in the type X collagen gene. Collagen X is a short, nonfibrillar collagen expressed by hypertrophic chondrocytes in the growth plates of long bones. MCDS patients suffer from a relatively mild chondrodysplasia characterized by growth plate malfunction with a strong expansion of the hypertrophic zones [58]. Almost all patients with MCDS carry mutations in the NC1 domain of type X collagen [37]. Tsang et al. generated transgenic mice carrying a disease-causing 13 base pair deletion (13del) in this domain [59]. These mice display a chondrodysplasia phenotype including short limbs and expanded hypertrophic zones in growth plates of long bones. Collagen X is retained in the ER cisternae of hypertrophic chondrocytes, and UPR marker proteins such as BiP, XBP1s, CHOP, and processed ATF6 are upregulated, suggesting MCDS to be an ER stress-related skeletal disease. Furthermore, the authors described changes in the chondrocyte differentiation program as part of the adaptive response to the ER stress. The hypertrophic chondrocytes are reprogrammed to a “prehypertrophic chondrocyte-like” cell showing proliferative characteristics to circumvent the expression of mutated collagen X. These aberrations from the normal differentiation processes during endochondral ossification then lead to the chondrodysplasia phenotype [59].

In all of these mouse models, mutations in ECM proteins result in the synthesis of misfolded proteins, which accumulate in the ER and induce the UPR. However, due to the intracellular retention of the mutated proteins, the ECM also lacks essential constituents or contains minor amounts of the affected ECM macromolecules. Therefore, it is hard to determine whether the ER stress itself, the loss of essential ECM components, or both is the underlying mechanism of the given chondrodysplasias.

### 5.2. Transgenic Mice with Mutations in Genes Encoding Exogenous Proteins That Are Normally Not Expressed in Cartilage

In order to analyze the effects of ER stress on bone development and long bone growth without loss of a single, essential cartilage ECM constituent, transgenic mice were generated in which mutated thyroglobulin (Tg^cog^) is expressed in chondrocytes [60, 61]. The mutated thyroglobulin fails to be folded, accumulates in the ER, and induces ER stress. However, as thyroglobulin normally is only expressed in the thyroid gland, the cartilage of these transgenic mouse lines does not lack any specific essential cartilage component.

#### 5.2.1. Tg^cog^ Mouse (Col2a1-Tg^cog^)

This mouse line was generated to investigate the generic role of ER stress and the UPR in the pathogenesis of the chondrodysplasia types MED and PSACH. Tg^cog^ was expressed as potential ER stress-inducing protein in proliferative chondrocytes under the control of the collagen II alpha 1 promoter. Due to its mutation, Tg^cog^ was retained in the ER cisternae, induced ER stress, and activated the UPR. This was detected by increased expression of the ER stress marker protein BiP, phosphorylation of eIF2*α*, and appearance of XBP1s, the spliced form of XBP1. Col2a1Tg^cog^ mice displayed diminished long bone growth and a reduced rate of chondrocyte proliferation. However, morphology of the chondrocytes and architecture of the overall growth plate were normal. In addition, no increased apoptosis was detectable. Summarized, these data demonstrate that the targeted induction of ER stress in chondrocytes is sufficient to reduce the rate of bone growth and establishes that classical ER stress is a pathogenic factor that contributes to the disease mechanisms of MED and PSACH. However, as not all pathological features of MED and PSACH were recapitulated, a combination of intra- and extracellular factors is suggested to be responsible for disease pathology [60].

#### 5.2.2. Tg^cog^ Mouse (Col10a1-Tg^cog^)

An analogous mouse model was established to examine the role of ER stress and the UPR in the pathogenesis of MCDS. Mutant thyroglobulin (Tg^cog^) was expressed in hypertrophic chondrocytes under the control of the collagen X promoter. The hypertrophic chondrocytes in these mice exhibited ER stress with a characteristic UPR response. In addition, the hypertrophic zone was expanded, gene expression patterns were disrupted, osteoclast recruitment to the vascular invasion front was reduced, and long bone growth decreased. Moreover, hypertrophic chondrocytes regain a prehypertrophic differentiation state comparable to chondrocytes of the MCDS mice. These data demonstrate that triggering ER stress in hypertrophic chondrocytes per se is sufficient to induce the essential features of the cartilage pathology associated with MCDS and confirm that ER stress is a central pathogenic factor in the disease mechanism [61].

Both Tg^cog^ mouse models prove that ER stress is centrally involved in the pathogenesis of chondrodysplasias. The advantage over mouse models with mutant ECM proteins is obvious, but the contribution of a possibly reduced concentration of numerous ECM components cannot clearly be ruled out as a potential factor in the pathogenesis of chondrodysplasias, as the induced UPR reduces the overall protein synthesis in affected chondrocytes. This is the reason why additional mouse models are important to confirm the direct link between ER stress and the pathogenesis of chondrodysplasias.

### 5.3. Mice with a Knockout of Genes Encoding Proteins of the ER Folding Machinery

To further characterize the role of ER stress as a pathogenic factor in chondrodysplasia, transgenic mice were generated with a cartilage-specific deficiency in proteins of the folding machinery or missing UPR signaling factors.

#### 5.3.1. Hsp47 KO Mouse (Col2a1 Hsp47 KO)

The most prominent knockout mouse model in this respect is the cartilage-specific Hsp47 KO mouse [38]. Masago et al. generated a mouse model with a conditionally inactivated Hsp47 gene in chondrocytes using Hsp47 floxed mice and mice carrying a chondrocyte-specific Col2a1-Cre transgene. Hsp47 binds Yaa-Gly-Xaa-Arg-Gly in triple-helical procollagen in the ER via hydrophobic and hydrophilic interactions. In cartilage, the binding of Hsp47 mainly stabilizes procollagen II by preventing unfolding of the triple helix. Thus, Hsp47 is crucial for efficient secretion, processing, fibril formation, and deposition of collagen type II in the ECM of cartilage [62]. These mice die just before or shortly after birth and exhibit a severe generalized chondrodysplasia and bone deformities with lower levels of type II and type XI collagen. Most long bones were severely twisted and shortened. First, these results demonstrate that Hsp47 is indispensable for well-organized cartilage fibril formation and normal endochondral bone formation. In addition, this mouse model displays numerous characteristics of ER stress. Cartilage collagens and other ECM components accumulated in the rough ER and induced the UPR with induction of the ER stress marker BiP. Moreover, the TUNEL assay revealed an elevated apoptosis rate. This ER stress induced chondrocyte death clearly contributes to the chondrodysplasia phenotype of cartilage-specific Hsp47 KO mice.

#### 5.3.2. ERp57 KO Mouse (Col2a1 ERp57 KO)

In our lab, the cartilage-specific ERp57 knockout mouse was intensively investigated [63]. ERp57 is a member of the protein disulfide isomerase family of ER chaperone proteins and is essentially involved in the formation of disulfide bridges in newly formed glycoproteins [21]. Moreover, ERp57 accounts for a folding correction in misfolded proteins by elimination of disarranged and formation of new disulfide bridges [64]. The activity of ERp57 is dependent on interactions with calnexin and calreticulin, which mediate the recognition and binding of N-glycosylated substrates [20, 65]. As a total deficiency of ERp57 in mice is lethal at embryonic day 13.5, demonstrating that ERp57 action is indispensable for vertebrate development [66], a cartilage-specific KO mouse model was generated. We used ERp57 floxed mice and crossed these with Col2a1-Cre transgenic animals [63]. The cartilage-specific ERp57 KO animals (ERp57cKO) display an obvious chondrodysplasia-like bone phenotype. Four-week-old male mice reveal a reduced weight, shorter long bones, enlarged growth plates, and a decreased proliferation and increased apoptotic cell death in growth plate chondrocytes. Most likely, this bone phenotype is pronounced especially at this age because of an extremely high protein demand due to the pubertal growth spurt. The significantly reduced tibia lengths and the enlarged growth plates were confirmed by *μ*-computer tomography analysis. By this method, also altered trabecular structures and a reduced bone volume compared to the total volume were detectable. With respect to the function of ERp57 in protein folding, chondrocytes were analyzed by electron microscopy. Dilated ER structures, which are most likely a consequence of accumulating misfolded proteins and therefore represent a sign of ER stress in these cells, were evident. Immunofluorescence staining of chondrocytes of the tibial growth plate indeed displayed higher amounts of the ER stress markers BiP and CHOP. This demonstrates that ER stress, triggered by the knockout of the protein disulfide isomerase ERp57, is the basic reason of the chondrodysplasia phenotype in these mice [63]. Therefore, mice with a cartilage-specific knockout of the protein disulfide isomerase ERp57 qualify as a novel model for the analysis of ER stress in chondrocytes and ER stress-related skeletal diseases. We wondered that the knockout of this single PDI led to this pronounced phenotype and started to examine ERp57 substrates. In preliminary studies, we observed that the secretion of collagen II by ERp57 KO chondrocytes was almost normal, whereas the proteoglycans were diminished as seen by alcian blue staining of micromass-cultured cells. These analyses, however, are far from accurate and should be expanded in future. Jessop et al. analyzed ERp57 substrates by biochemical means and found common structural domains which are important for the interaction of ERp57 with the proteins to be folded [67]. These specific domains may also be one reason why other protein disulfide isomerases, such as PDI or ERp72, could not efficiently compensate for the loss of ERp57. The superior function of the calreticulin-calnexin ERp57 cycle over other protein folding mechanisms [20] might also play a role in this context. The complex of multiple proteins that work together is important for speeding up the folding process. If the calreticulin-calnexin-ERp57 cycle fails, this cannot be compensated by individual PDIs. These results and additional detailed analyses of the ERp57 KO mouse in the future will provide access to new insights into ER stress-related cartilage diseases.

As these mice also display chondrodysplasia-like phenotypes, ER stress gets more and more likely as a pathogenic factor in chondrodysplasia.

### 5.4. Mice with a Knockout of Genes Encoding Proteins Influencing UPR Signaling

ER stress initiates the UPR to restore cellular homeostasis by expression of additional chaperones, reduction of translation, and initiation of the degradation of aggregated proteins. Whenever the UPR fails or is not able to counteract the ER stress, cellular homeostasis cannot be restored and apoptosis is initiated. In mice with deficiencies in UPR signaling factors, this restoration system is reduced, and consequently, the ER stress remains high for a longer period. Therefore, the initiation of pathologic outcomes is anticipated. However, if only one UPR signaling route is blocked, the others probably can substitute for this loss. Here, different mouse models with a failure in ER stress-induced signaling processes in chondrocytes are described exemplarily.

#### 5.4.1. XBP1 KO Mouse (Col2a1-XBP1 KO)

In presence of aggregated proteins, the ER sensor protein IRE1*α* is activated and then induces splicing of XBP1. The spliced form of XBP1 (XBP1s) acts as a transcription factor inducing the expression of different genes involved in quality control mechanisms of the ER. Cartilage-specific XBP1 knockout mice [39] display a mild form of chondrodysplasia with a delay in endochondral ossification. The main characteristics are dysregulation of chondrocyte proliferation and shortening of hypertrophic growth plate zones. Moreover, long bones reveal a delayed ossification. While ER stress was enhanced in the *XBP1*-deficient growth plate cartilage and was detectable by IRE1 hyperactivation, only minimal alterations in the expression of chondrocyte proliferation markers were observed and no changes in apoptotic cell death were detectable. The effects of a XBP1 deficiency in cartilage are rather low, but even small imbalances in chondrocytes induce changes in the timing of mineralization during endochondral ossification, ending in an ER stress-induced chondrodysplasia phenotype [39].

#### 5.4.2. ATF4 KO Mouse (Total KO)

ATF4 positively regulates the expression of UPR genes that are involved in folding and regulation of autophagy and apoptosis, e.g., CHOP and GADD34 [32]. Compared to XBP1 deficiency, the ablation of *Atf4* in mice leads to more severe skeletal defects. ATF4-deficient mice display a 50% reduction in body weight and in femoral bone length at the age of 1 month, indicating a severe limb dwarfism. In the growth plates, the typical columnar structure of proliferative chondrocytes is disturbed and the proliferative zone is shortened. In addition, the hypertrophic zone is abnormally expanded, suggesting a delay in the overall endochondral ossification process. Detailed analysis in chondrocytes showed that ATF4 acts as a transcriptional activator of Indian Hedgehog and therefore controls chondrocyte proliferation and differentiation during bone development and growth [40].

#### 5.4.3. BMP2 KO Mouse (Col2a1-Bmp2 KO)

During bone development by endochondral ossification, BMP2 activates via Smad-4 the transcription of XBP1, which is involved in ER stress signaling and positively regulates bone formation by activating granulin-epithelin precursor [68]. Both is essential, as mice with a conditional knockout of BMP2 develop a severe chondrodysplasia, with defects in proliferation, differentiation, and apoptosis of chondrocytes in the growth plate [69]. The phenotypes of cartilage-specific BMP2 and XBP1 KO mice are comparable.

#### 5.4.4. *S1P* KO Mouse (Col2a1-*S1P*)

The site 1 protease is essential in UPR signaling as well, as it is responsible for the processing and activation of the transcription factor ATF6. Due to a loss of active ATF6, one of the three UPR signaling pathways is completely shut down, and therefore, S1P KO mice exhibit a severe chondrodysplasia with a substantial increase in chondrocyte apoptosis. Ultrastructural analysis revealed that the ER in S1P KO chondrocytes displays characteristics of severe ER stress. This suggests that S1P activity is required for the genesis of normal cartilage by endochondral ossification [70].

These mouse models clearly demonstrate that UPR signaling factors play a role not only under pathological but also under physiological conditions in cartilage. Without a well-functioning ER quality control system, normal endochondral ossification and regular bone growth are disturbed.

### 5.5. Mice with a Knockout of Proteins Involved in the Degradation of Aggregated Proteins

In addition to other processes, the UPR initiates the degradation of aggregated proteins by ER-associated degradation (ERAD type I) or autophagy (ERAD type II). These mechanisms are commenced to liberate the chondrocytes from the overflow of unfolded or misfolded proteins in the ER. However, if the translocation processes into the cytosol or the degradation processes fail, the ER stress in affected chondrocytes remains and the initiation of apoptosis is the last chance to rescue cartilage homeostasis.

#### 5.5.1. Derlin-2 KO (Total KO)

A multiprotein complex linking dislocation, ubiquitination, and extraction of misfolded proteins from the ER membrane mediates the dislocation process from the ER to the cytosol in the case that misfolded proteins should be degraded in the proteasome [71]. Derlin-2 is one of the important players within this multiprotein complex. Whole-body deletion of derlin-2 in mice results in perinatal death of most of the animals due to feeding failures. However, the few mice that survive to adulthood develop a severe chondrodysplasia with defects in ECM protein synthesis and secretion. Derlin-2 KO mice were smaller, had reduced bone lengths, and displayed a striking involution of the rib cages with fusion of the third and fourth sternebrae. Chondrocytes of KO animals showed intracellular retention of ECM components, suggesting a defective protein secretion and degradation. In addition, higher CHOP RNA and protein levels suggest an increase in chondrocyte apoptosis in these animals [72].

#### 5.5.2. CTGF/CCN2 (Total KO)

In addition to ERAD type I, autophagy (ERAD type II) is activated in case of ER stress. This mechanism is of great importance for the functionality and homeostasis of the growth plates in mice, as the loss of the connective tissue growth factor (CTGF/CCN2), which is a positive regulator of autophagy, leads to a severe chondrodysplasia. CTGF/CCN2 KO chondrocytes displayed dilated ER cisternae, increased apoptosis, and increased BiP and CHOP expression levels [73]. Overall, autophagy is considered as a particularly important mechanism for the survival of highly secretory cells under hypoxic and other stressful conditions [74].

The occurrence of chondrodysplasias due to the loss of proteins engaged in ERAD I or II demonstrates the importance of a functional degradation system for unfolded or misfolded proteins. A retention of aggregated proteins in the ER induces ER stress in chondrocytes culminating in dysfunction of the skeletal system.

### 5.6. Mice with a Knockout of Proteins Which Are Essential for Protein Trafficking and Secretion

In addition to the abovementioned processes, disturbed trafficking of proteins within the cells and to the cell surface and insufficient secretion induce skeletal phenotypes as well.

#### 5.6.1. BBF2H7 KO Mouse (Total KO)

Under normal conditions, ER stress induces the activation of the transcription factor BBF2H7, which then, via Sec23, encodes a component that is responsible for the protein transport from the ER to the Golgi apparatus. If BBF2H7 and subsequently Sec23 are absent, the formation of vesicles for these transport processes fails and cartilage matrix secretion is impaired. BBF2H7 KO mice showed severe chondrodysplasia and died by suffocation shortly after birth because of an immature chest cavity. ECM proteins, type II collagen, and COMP remained in the ER; ER stress arose and a severe chondrodysplasia with characteristics, such as a lack of the typical columnar structure in the proliferating zone of growth plates and proliferating chondrocytes showing abnormally expanded ERs developed. This indicates that the ER stress-induced BBF2H7-Sec23a pathway is crucial in endochondral ossification as insufficient protein trafficking into the ECM results in ER stress counteracting proper cartilage proliferation and differentiation processes [75]. In addition, a C-terminal fragment of BBF2H7 accelerates chondrocyte proliferation by binding to Indian hedgehog and its receptor PTCH1, supporting their interaction and signaling [76]. If this acceleration is missing, the proliferative capacity of chondrocytes is reduced.

#### 5.6.2. GMAP-210 KO Mouse (Col2a1-Trip11 KO)

Another trafficking component of interest is the golgin Golgi microtubule-associated protein of 210 kDa (GMAP-210). Like other golgins, GMAP-210 is required for efficient trafficking from the ER to the Golgi. Studies by Bird et al. revealed that the skeletal phenotype of achondrogenesis type 1A is exclusively caused by defects in chondrocytes, but not osteoblasts, osteoclasts, or hematopoetic cells. Loss of GMAP-210 led to a massive ER swelling and cell death in growth plate cells culminating in impaired bone formation. Notably, the intracellular accumulation of proteins applies only to specific ECM components such as perlecan, but is not a general secretion defect [77].

These examples show that mice with trafficking defects display ER stress. Consequently, it seems very likely that chondrodysplasias in mice with such defects are not induced by the loss of nonsecreted components in the ECM, but by the increasing ER stress in chondrocytes.

## 6. The Skeletal Phenotypes of Different ER Stress Mouse Models Show a High Degree of Similarity

**Figure 2:**
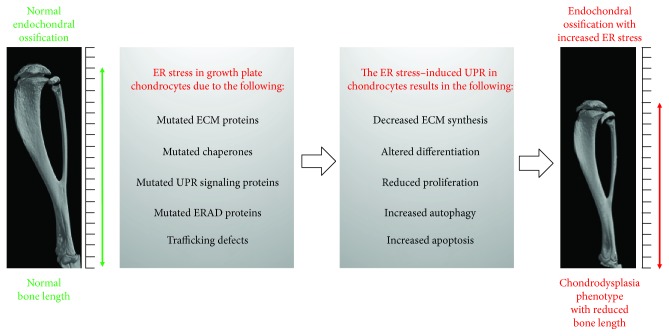
Causes and effects of ER stress in growth plate chondrocytes. ER stress in growth plate chondrocytes is induced by mutations in genes encoding ECM components, chaperones, UPR signaling factors, or ERAD proteins. The ER stress-induced unfolded protein response (UPR) substantially impairs essential processes of the endochondral ossification, such as ECM protein synthesis and chondrocyte proliferation and differentiation, and activates autophagy and apoptosis. This results in a chondrodysplasia phenotype with reduced lengths of long bones.

Taken together, various mouse models with prolonged or high levels of ER stress in growth plate chondrocytes display chondrodysplasia phenotypes (Figure 2). However, the cause for this pathological outcome varies between the different ER stress mouse models. ER stress may arise due to misfolding of ECM proteins, failures in the ER folding machinery, malfunction of UPR signaling factors, or mutations in proteins involved in the degradation of aggregated proteins. All these cellular dysfunctions result in high levels of ER stress and are obvious, e.g., by dilation of ER cisternae due to the accumulation of unfolded or aggregated proteins. The cells react via UPR by a reduction of protein synthesis, an induction of additional folding enzymes, and by degradation of aggregated proteins. If these rescue mechanisms succeed, chondrocytes regain their normal function. If not, ECM protein synthesis is reduced for a longer period of time, proliferation decreases, differentiation of chondrocytes gets impaired, and apoptosis is augmented. All these changes critically impact bone development.

There are several essential steps in the endochondral ossification process and even slight imbalances at one or the other checkpoint affect the overall process. Long bone growth can be inhibited due to an ER stress-related reduced protein synthesis of cartilage ECM components by affected chondrocytes. The diminished ECM then leads to a reduced bone volume and manifests in a dwarfism disease. Another feature is an ER stress-induced change in chondrocyte proliferation or enhanced apoptosis. Together, this leads to a reduced number of chondrocytes in the growth plates affecting the overall process of endochondral ossification. In addition, ER stress-induced changes in the differentiation program of chondrocytes during endochondral ossification lead to dwarfism diseases. If only one of these necessary events fails, a normal bone development is impossible. This demonstrates that a finely tuned chondrocyte homeostasis with a distinct competence to cope with ER stress in all growth plate zones is essential for a regulated bone development and long bone growth.

## 7. ER Stress Also Affects Degenerative Cartilage Diseases Such As Osteoarthritis

ER stress, however, does not only affect temporary cartilage, which is involved in skeletal development. The function of permanent cartilage in various joints also is dependent on a regulated protein synthesis without enhanced ER stress levels. If this is altered, degenerative cartilage diseases, such as osteoarthritis (OA), may arise. OA is most likely associated with changes in protein synthesis and changes in proliferation and differentiation of chondrocytes and enhanced apoptosis. These OA features are similar to ER stress-induced characteristics occurring during bone endochondral ossification.

In general, OA preferentially develops in the elderly. However, chondrodysplasias are often associated with cartilage degradation in young patients [37, 78]. Stickler syndrome, for example, is a dominantly inherited vitreoretinopathy and chondrodysplasia caused by mutations in the genes for types II and XI collagen [79, 80]. About 50% of all stickler syndrome patients develop severe cartilage erosion in diarthrodial joints before they get 30 years old, and this may be the result of ER stress in articular chondrocytes [81].

In the literature, various studies describe a link between osteoarthritis and ER stress [82–89]. During OA chondrocytes undergo activation, they proliferate and form cell clusters. In addition, the protein synthesis of both ECM molecules and matrix-degrading proteases increases [90], and thus, a high protein load arises in the ER. In late stages of OA, however, cartilage cells reduce their proliferation and die by apoptosis. With age, the expression and activity of folding enzymes for the ER and the UPR signaling decline [91]. Thus, an increased protein synthesis during OA and a weaker UPR response may induce ER stress, which cannot be compensated [92]. Moreover, the concentration of advanced glycation end products (AGEs) increases and AGEs are known to induce ER stress signaling [93, 94]. We assume that ER stress may contribute to the development of OA in young chondrodysplasia patients as well as in aged individuals.

In chondrocytes of osteoarthritis patients compared to cartilage cells of healthy controls, a 1.5-fold increase of ERp57 was detected. Simultaneously, calnexin and calreticulin were induced and the stimulation with thapsigargin, an ER stress inducer, increased the ERp57 expression in chondrocytes 2.8-fold [84]. Accordingly, ERp57 and the calnexin/calreticulin cycle appears to be particularly active during ER stress in osteoarthritic cartilage cells. We suggest to employ the cartilage-specific ERp57 KO mouse for a detailed analysis of the contribution of ER stress in the pathogenesis of different skeletal diseases, including OA.

## 8. Outlook/Conclusion

Various mouse models with prolonged or high levels of ER stress in growth plate chondrocytes display chondrodysplasia phenotypes suggesting ER stress as a pathogenic factor contributing to these skeletal diseases. We analyzed in detail the phenotype of the cartilage-specific ERp57 knockout mouse and demonstrated that the deficiency of this single ER-resident protein disulfide isomerase, which is responsible for the correct building of disulfide bridges in cartilage ECM glycoproteins, is sufficient to induce ER stress in chondrocytes and to cause an ER stress-related bone phenotype resembling a chondrodysplasia. This mouse line therefore qualifies as a novel model for the analysis of ER stress in chondrocytes. It may give new insights in ER stress-related short stature disorders and enables the analysis of the role of ER stress in other cartilage diseases, such as osteoarthritis. By proteomic analysis, it would be possible to examine whether specific or all ECM proteins accumulate in the ER of ERp57 cKO mice. Comparable tests in different MED and PSACH mouse models with ER stress revealed genotype-specific differences, suggesting common and distinct disease signatures [95]. This shows that ER stress in different mouse models may have diverse effects. Therefore, multiple ER stress mouse models are valuable to examine cartilage diseases in further detail and to test therapeutics to intervene. Possible therapeutic options are as follows: (1) small molecular chemical chaperones, which efficiently support protein folding in the ER and ameliorate UPR signaling; (2) inhibitors of single UPR pathways, such as PERK signaling inhibitors [96]; (3) activators of ER-associated degradation and autophagy, such as carbamazepine [97]; or (4) inhibitors of excessive apoptosis. However, it is further to be tested whether such compounds alone or in combination are applicable to achieve improvement in ER stress-related cartilage disorders in patients.

## Figures and Tables

**Table 1:** Skeletal phenotypes in mice with ER stress in chondrocytes.

	ERp57 KO	MED	PSACH	PSACH	MCDS	Chondrodysplasia	SED	Tg^cog^	Tg^cog^	Hsp47 KO	XBP1 KO	ATF4 KO	SP1 KO	BMP2	Derlin-2	CTGF/CCN2	BBF2H7 KO	GMAP-210 KO
Reference	[63]	[49]	[53]	[57]	[59]	[43]	[45, 46]	[61]	[60]	[38]	[39]	[40]	[70]	[69]	[72]	[73]	[75]	[77]
Genetic modification	Col2a1-ERp57 KO	p.Val194Asp in *MATN-3*	p.Thr583Met in *COMP*	p.Asp469del in *COMP*	13del in *COL10a1*	p.Gly1170Ser in *Col2a1*	p.Asp1192Cys in *Col2a1*	Col10a1-Tg^cog^	Col2a1-Tg^cog^	Col2a1-Hsp47 KO	Col2a1-XBP1 KO	Atf4 KO	Col2a1-SP1 KO	Col2a1-Bmp2 KO	Der2 KO	Ccn2 KO	Bbf2h7 KO	Col2a1-Trip11 KO
Protein secretion	n/a	No secretion of targeted protein	Secretion of targeted protein	Reduced secretion of targeted protein	No secretion of targeted protein	Less col II secretion	Less col II secretion	No secretion of targeted protein	No secretion of targeted protein	Less col II and XI secretion	Normal secretion of Col II and Col X	Normal secretion of Col II	Less col II secretion	n/a	Retention of collagen matrix proteins	Fewer collagen fibrils in embryos E18.5	Less Col II and COMP secretion	Less perlecan secretion
Weight	↓−25% at the age of 4 wks	↓−9.5% at the age of 9 wks	↓−6% at the age of 9 wks	↓ -6% at the age of 6 wks	n/a	No weight changes during embryogenesis	↓−30% at the age of 6 wks	n/a	↓At the age of 3, 6, and 9 wks	↓−17% in embryos E18.5	n/a	↓−50% at the age of 4 wks	n/a	n/a	↓In neonates	n/a	n/a	n/a
Bone length	↓−14% in the tibia at the age of 4 wks	↓−12.5% in the tibia at the age of 3 wks	↓−4% in the tibia at the age of 9 wks	−6% in the femur at the age of 9 wks	↓−15% in the tibia at the age of 10 wks	↓During embryo-genesis and in neonates	↓At the age of 10 wks	↓−6% in the tibia at the age of 6 wks	↓−2, 3, and 7% in the femur at the age of 3, 6, and 9 wks	↓In embryos E15.0 and E18.5	↓−13% in tibia and femur at the age of 2 wks	↓−50% in the femur at the age of 4 wks, ↓humerus in embryos	↓All skeletal elements in neonates	↓All skeletal elements in embryos E18.5	↓In neonates	n/a	↓In embryos E18.5	↓In embryos E17.5
Changes in the GP, PZ, or HZ	↑GP, ↑HZ at the age of 4 wks	Disorganized GP at the age of 1 and 3 wks	↑PZ, disorganized GP at the age of 3 wks	Disorganized GP at the age of 3 wks, areas of hypocellularity in PZ	↑HZ at the age of 10 d	Disorganized GP, loss of HZ in embryos E19.5	Disorganized GP, cell-free areas at the age of 6 and 10 wks	↑HZ in neonates and at the age of 3 wks	Normal	n/a	Hypocellular areas, ↑PZ, ↓HZ (−45%), more cells per column at the age of 2 wks	↑HZ, disorganized GP in embryos E16	Differentiation into hypertrophic cells disturbed	↓PZ, ↓HZ, disorganized HZ in embryos E18.5	n/a	↓HZ in neonates	Lack of columns in PZ, decreased number and size of cells in HZ	↓HZ in embryos E15.5
ER structure	Dilated cisternae in neonates	Dilated cisternae at the age of 7 d	n/a	Dilated cisternae at the age of 7 d	Dilated cisternae at the age of 10 d	Dilated cisternae in embryos E19.5	n/a	n/a	n/a	Dilated cisternae in embryos E18.5	n/a	n/a	Dilated cisternae in embryos E18.5	n/a	Dilated cisternae in embryos E18.5	Dilated cisternae in embryos E18.5	Dilated cisternae in embryos E18.5	Dilated cisternae in embryos E15.5
UPR marker expression	↑BiP and CHOP at the age of 4 wks	↑BiP and Grp94 at the age of 3 d	↑BiP, Crt, CHOP, phosphorylated eIF2*α*, processed ATF6 at the age of 3 wks	Normal	↑BiP, XBP1s, EDEM, CHOP at the age of 10 d	↑CHOP, XBP1, XBP1s, BiP, ATF4, ATF6 in neonates	↑BiP at the age of 2 wks	↑BiP, processed ATF6 at the age of 3 wks	↑BiP, phosphorylated eIF2*α* at the age of 3 wks, XBP1s at the age of 5 d	↑BiP in embryos E18.5	↑BiP, IRE1 at the age of 3 d	n/a	n/a	n/a	↑CHOP	↑BiP, CHOP, calnexin in embryos E18.5	↑BiP, PDI, GRP94, ATF4 in embryos E18.5	n/a
Proliferation	↓At the age of 4 wks	↓At the age of 3 wks	↓At the age of 3 wks	↓At the age of 3 wks	n/a	↓In embryos E18.5	↓In neonates and at the age of 10 wks	n/a	↓At the age of 3 wks	n/a	↓At the age of 2 wks	↓In embryos E16 and in neonates	Normal	↓In embryos E18.5	n/a	↓In embryos E16.5	n/a	n/a
Apoptosis	↑At the age of 4 wks	↑At the age of 3 wks	↑At the age of 3 wks	↑At the age of 3 wks	Normal	↑In embryos E18.5	n/a	n/a	Normal	↑In embryos E18.5	Normal	↑In neonates	↑In embryos E15.5	↑In embryos E18.5	n/a	↑In embryos E18.5	Normal	n/a
Osteoarthritis	Under investigation	n/a	Spontaneous OA at the age of 16 mo	n/a	n/a	n/a	n/a	n/a	n/a	n/a	n/a	n/a	n/a	n/a	n/a	n/a	n/a	n/a

n/a = not analyzed, GP = growth plate, PZ = proliferative zone, HZ = hypertrophic zone, Col = collagen, E = embryonal stage, ↑ = increased, ↓ = reduced, d = days, wks = weeks, and mo = months.
